# Supplementary material for: Causal Relationship between Aging and Anorexia Nervosa: A White-Matter-Microstructure-Mediated Mendelian Randomization Analysis
Source: Biomedicines. 2024 Aug 16;12(8):1874. doi: 10.3390/biomedicines12081874 (PMC11351342; doi:10.3390/biomedicines12081874)
Supplement: Supplementary file 1 [file biomedicines-12-01874-s001.zip › Supplementary Figure/Figure S8/Leaveoneout plot of MR analysis for TL to ubm-b-1984.pdf]

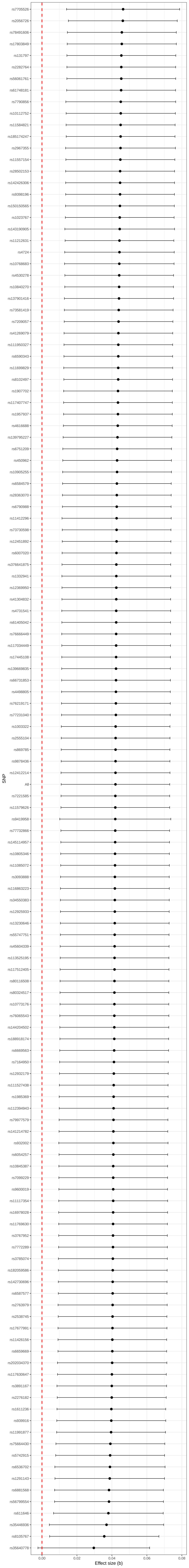
rs7705526
rs2056726
rs78491606
rs17803849
rs131797
rs2282764
rs56061761
rs61748181
rs7790856
rs10112752
rs11584821
rs185174247
rs2967355
rs11557154
rs28502153
rs142426306
rs9398196
rs150150565
rs1023767
rs143190905
rs11212631
rs4724
rs10768683
rs4530278
rs10840270
rs137901416
rs73581419
rs7209057
rs41269079
rs111950327
rs6590343
rs11699829
rs8102497
rs1907702
rs117407747
rs1957937
rs4616688
rs139795227
rs6751209
rs450962
rs10905255
rs6584579
rs28363070
rs6790988
rs11412296
rs73730598
rs12451892
rs6007020
rs376641875
rs1332941
rs12369950
rs41304832
rs4731541
rs61405042
rs76666449
rs117034449
rs17445108
rs139669835
rs66731853
rs4498805
rs76219171
rs77231040
rs1003322
rs2555104
rs869785
rs9878436
rs12412214
All
rs7221585
rs11579626
rs9419958
rs77732866
rs145114957
rs10805346
rs11085072
rs3093888
rs116863223
rs34550383
rs12925933
rs13230646
rs55747751
rs45604339
rs113525195
rs117512405
rs80116508
rs80324517
rs10773176
rs76065543
rs144204502
rs188918174
rs6669563
rs7164950
rs12932179
rs111527438
rs1985369
rs112394943
rs79977579
rs141214782
rs932002
rs6054257
rs10845387
rs7099229
rs9600019
rs11117354
rs16978028
rs11769630
rs3767952
rs7772289
rs3785074
rs182059586
rs142730696
rs6587577
rs2763979
rs2538745
rs17677991
rs11426156
rs6659669
rs202034370
rs117630647
rs3891167
rs2276182
rs1611236
rs939916
rs11991877
rs75664430
rs5742915
rs6536702
rs1291143
rs6881568
rs56799554
rs611646
rs35446936
rs8105767
rs35640778
SNP
0.00
0.02
0.04
0.06
0.08
Effect size (b)
